# 'Brukin2D': a 2D visualization and comparison tool for LC-MS data

**DOI:** 10.1186/1471-2105-10-S6-S12

**Published:** 2009-06-16

**Authors:** Dimosthenis Tsagkrasoulis, Panagiotis Zerefos, George Loudos, Antonia Vlahou, Marc Baumann, Sophia Kossida

**Affiliations:** 1 Protein Chemistry/Proteomics Laboratory and the Neuroscience Research Program Biomedicum Helsinki P.O. Box 63 (Haartmaninkatu 8) 00014 University of Helsinki, Finland; 2 Bioinformatics & Medical Informatics Team, Biomedical Research Foundation of the Academy of Athens, 11527 Athens, Greece; 3 Biotechnology Division, Proteomics Unit, Biomedical Research Foundation of the Academy of Athens, 11527 Athens, Greece

## Abstract

**Background:**

Liquid Chromatography-Mass Spectrometry (LC-MS) is a commonly used technique to resolve complex protein mixtures. Visualization of large data sets produced from LC-MS, namely the chromatogram and the mass spectra that correspond to its compounds is the focus of this work.

**Results:**

The in-house developed 'Brukin2D' software, built in Matlab 7.4, which is presented here, uses the compound data that are exported from the Bruker 'DataAnalysis' program, and depicts the mean mass spectra of all the chromatogram compounds from one LC-MS run, in one 2D contour/density plot. Two contour plots from different chromatograph runs can then be viewed in the same window and automatically compared, in order to find their similarities and differences. The results of the comparison can be examined through detailed mass quantification tables, while chromatogram compound statistics are also calculated during the procedure.

**Conclusion:**

'Brukin2D' provides a user-friendly platform for quick, easy and integrated view of complex LC-MS data. The software is available at .

## Background

Proteomics is the large scale analysis of proteins in a biological system. It is a continuously and rapidly growing scientific area evolving together with the emergence of new methodologies and technologies. Protein identification is commonly being performed by the so called "bottom-up" approach: Proteins are converted to peptides, the sequences of which are then determined by tandem mass spectrometry (MS-MS), combined with the use of search engines and protein databases that are available in the internet [1]. This analysis is widely performed by the use of Liquid Chromatography-Mass Spectrometry (LC-MS). In a common implementation, a reversed-phase microcapillary LC system is coupled online with an electrospray ionization (ESI) tandem mass spectrometer. Peptides eluting from the LC column are immediately ionized by ESI and subjected to mass measurement (survey scan). The instrument, under computer control, automatically selects specific precursor ions for fragmentation in a collision cell, thus generating collision-induced dissociation (CID) and producing tandem mass spectra (MS/MS) [2,3]. It should be noted that many diverse chromatographic separations can be serially applied (multi-dimensional chromatography) and connected to the mass spectrometer, providing the ability to identify and quantify hundreds to thousands of proteins in a single experiment [4].

In the vast majority of computational biology aspects, the main challenges of LC-MS are handling, evaluating and visualizing the large data sets that are generated by the application of this technology. During a typical MS-based proteomics experiment, involving the analysis of protein fractions from cells, tissues or body fluids [5], thousands of mass spectra are generated. The use of mean mass spectra that correspond to the chromatogram compounds reduces dramatically that number; nevertheless the problem of quick viewing and evaluating the whole of these spectra is not trivial.

This paper is organized as follows: The implementation section presents the software in more detail, by describing the main ideas and functionality that are incorporated in it. This is followed by the results section, which summarizes the capabilities of the software by means of an application example. A brief comparison of other similar software tools is also included here. General remarks and future work are mentioned in the discussion section. The final section of the paper contains a summary of the conclusions.

## Implementation

In this work, we present an application, which provides a fast and easy way to overview a complete chromatograph analysis. 'Brukin2D' is a Matlab based program, which was developed in order to visualize, evaluate, process and compare LC-MS data. It uses as input the compound data that are exported from the Bruker 'DataAnalysis' program, and depicts the mean mass spectra of all the chromatogram compounds from one LC-MS run, in one 2D contour plot. Each spot in the plot represents one peptide mass. The spot's y-intercept determines the time of its compound in the chromatogram, while the x-intercept is the mass to charge ratio (m/z) of the corresponding peak. The darkness of the spots is proportional to the intensity of the mass peaks. Two contour plots from different chromatograph runs can be overlapped in the same window and compared. The results of the comparison can be viewed (and further processed) through detailed mass quantification tables and chromatogram compound statistics that are calculated during the procedure. In this communication, two protein mixtures are visualized and compared as an example of the main functionality features of this software tool.

'DataAnalysis' is an application created by Bruker Daltonics, which captures, visualizes and can further process the data that are produced from a LC-ESI-MS Bruker instrument. Some basic functionality features of 'DataAnalysis' include viewing the chromatogram, normalizing it and finding its compounds. Furthermore, it is capable of visualizing the MS or MS/MS spectra, removing the noise, deconvoluting the spectra and finding the mass peaks. Data can be extracted from the application in different formats (see Bruker 'DataAnalysis' application manual).

The 'Brukin2D' code and graphical user interface were written and tested in Matlab 7.4 for Windows XP 32bit and 64bit. The application (Matlab source code or Windows 32bit standalone application), its manual and the supplementary files that will be used in the application example below, can be freely downloaded from our website [6].

### Software description

The main window of the application is used in order to load the xml input files and plot the mass contours. The chromatogram and compound mass spectrum plots, along with the quantification data of the analysis are also provided here. The contour plots can be exported as image files. Finally the dual view window can be opened in order to compare two mixes.

**Figure 1:**
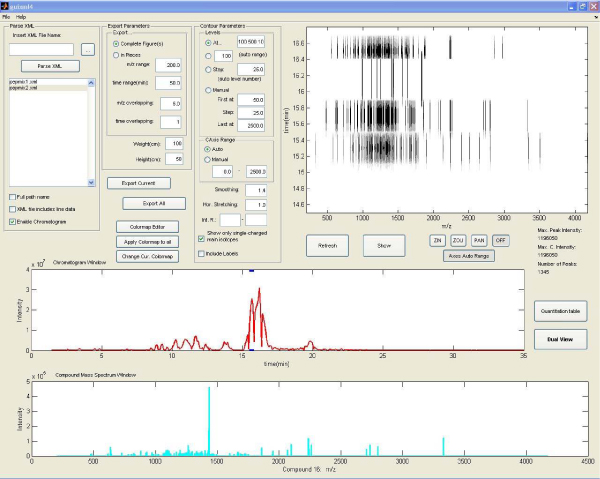
**'Brukin2D' main application window**. Parse XML, Export and Contour Parameters panels can be seen in the upper left corner. The main window at the right shows the contour plot. The red and cyan diagrams correspond to the chromatogram and the mass spectrum respectively.

The main window consists of different panels (figure 1). A short description of the functionality of these panels follows:

#### ParseXML panel

This panel is used to import and select the compound mass spectra files that have been exported from the Bruker 'DataAnalysis' program in xml format. Upon typing the file name and clicking on the parse XML button, the file is parsed and the contour plot is produced according to the inserted contour parameters (see below).

#### Contour Parameters panel

Each contour/density plot includes spots that correspond to mass peaks. Each spot consists of ellipsoid patches, which are drawn one above the other. These patches correspond to specific intensity contour levels and their color gets darker as the intensity gets bigger. This panel contains all the parameters that need to be set prior to contour plotting. For example, it is possible to adjust the number of the contour levels and each one's intensity value, the correspondence of color with intensity, the smoothing or stretching of spots, which is useful in order to acquire a clear and easy to evaluate plot. A detailed description of these parameters is included in the software manual.

#### Export panel

From this panel it is possible to export plots, complete or in parts, in jpeg or tiff format.

**Figure 2:**
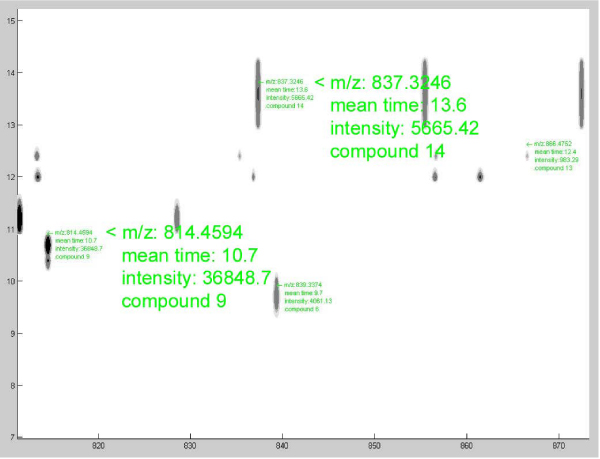
**Contour plot window**. Screenshot of a zoomed in region, with overlapping information for some of the mass peaks. This information can be viewed in an undocked plot window, by clicking on a spot of interest.

In addition, the main window incorporates three plot windows, which show the contour plot (figure 2), the chromatogram of the selected analysis and the compound mass spectrum of the selected compound. The quantification table includes detailed information for each mass peak in the plot and the dual view window allows the comparison of two mixes. Both can be accessed through the appropriate buttons in the main window.

**Figure 3:**
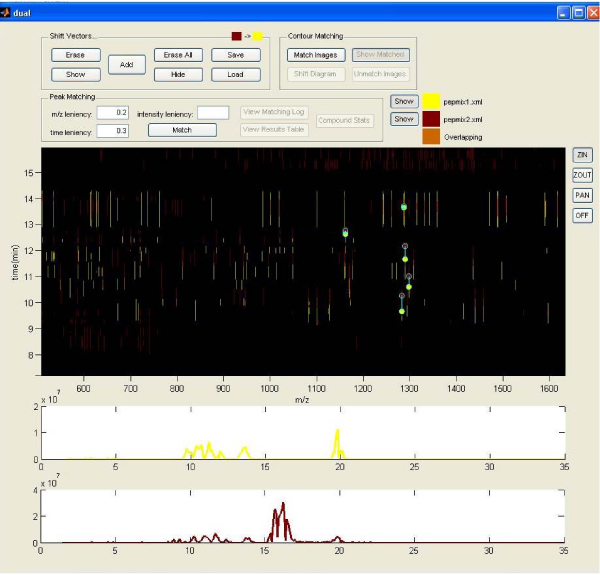
**Dual View window**. The two contour plots are shown superimposed in the center window. From there, we can enter the shift vectors in order to warp the two images. Below are the two corresponding chromatograms.

**Figure 4:**
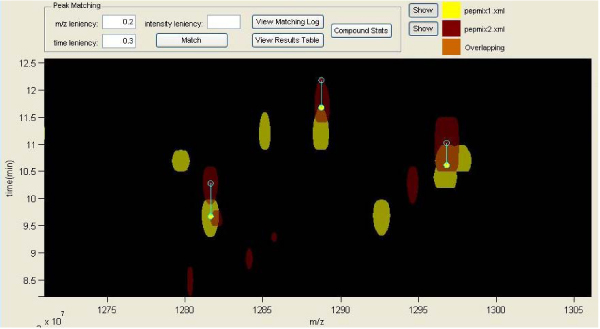
**Finding same mass peaks and inserting the appropriate shift vectors**. Only one shift vector is accepted per chromatogram compound.

We will discuss in more detail the functionality of the dual view window (figures 3 and 4), since it provides a semi-automated way to compare two contour plots, and find the differences between the two chromatograph runs. After clicking the dual view button in the main window, a dialog panel opens in order to select two mixes from the parsed ones. When the selection is made, the dual view window opens. It contains the two contour plots, which are superimposed, as well as the chromatograms of the mixes. A problem in LC-MS is that between different chromatograph runs of even the same peptide mix, there may be slight differences in the compounds' elution times (i.e. same compounds appear at slightly different times in the chromatograms). In order to eliminate these time differences, it is possible to insert shift vectors manually, through the dual view window. The application can then automatically compare the two plots and find the similarities and differences between the mass peaks, as well as the chromatogram compounds. There are parameters that can be set in order to control the leniency of that matching process. The results can be viewed through assimilated quantification tables and bar figures.

## Results

### Application example

This example demonstrates the main functionalities of the 'Brukin2D' program. Two peptide samples were used, which consist of the same basic peptide mix, with the addition of another standard mix in the second sample. The two samples were analyzed using LC-MS. Afterwards, the chromatogram compounds, their m/z spectra and the deconvolution data were calculated using the Bruker 'DataAnalysis' program. Finally, the compound data were exported in xml format, and also the chromatogram data in mzxml format.

The first processing step is to parse/analyze the xml files. While parsing, the application produces the chromatogram plot and the 2D contour visualization, according to the parameters that were selected from the contour parameters panel. The same parameter values are used for both mixes. In detail, four contour levels are selected in the intensity values 100, 500, 1000, 5000. Since the previous intensity values are relatively low, the smoothing value is set to 1.4, in order to acquire more rounded and consequently distinguishable peaks. Furthermore, the appropriate checkbox is marked, for the visualization to include only the deconvoluted m/z peaks. This means that all isotopes and multiple charged peaks are added together, so that only single-charged isotopes are shown in the plot. When the procedure is finished, some basic information can be seen in the main window. The first mix has 932 mass peaks in all the compounds, while the second has 1345. In addition to the chromatogram and the contour plot, the mean mass spectrum of each compound can be seen by clicking in the appropriate compound in the chromatogram. This can also be done by clicking in the contour window, within the time borders of that compound.

The mass matching and comparison of these two mixes is done by opening the dual view window. It must be mentioned that in the dual view window, only the lowest intensity level of the two contour plots is shown, due to time and memory issues. So, it is suggested for every comparison analysis to have at least the same lowest contour level for both mixes. The first step in comparing two mass contours is to eliminate any time differences produced by the LC-MS procedure. This is done by using vertical shift vectors, which are applied manually in the dual view window. By zooming and inspecting the overlapping contours and their chromatograms below, compounds or masses that are actually the same in the two mixes but appear in slightly different times in the analysis can be found. After inserting the appropriate vectors, the two contours are shifted in order to see the resulted images. The shift vectors have a red to yellow direction, so the second (red) mix is the one that is actually shifted to match the first (yellow) one (figure 4).

The next step, after shifting the contours, is to execute the peak matching procedure, during which, the application finds the matched peaks and creates combinatory quantification tables that give analytical information on each peak and its matched ones. The two parameters that have to be set here are the time and m/z leniency, which in the particular example were set to 0.3 and 0.2 respectively. These values actually define the maximum distances in the y (time) and x (m/z) axes that two matched peaks can have.

**Figure 5:**
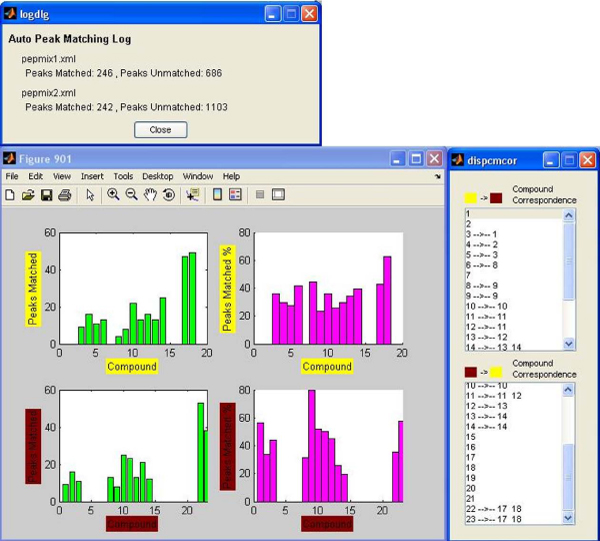
**Results of the matching process – no intensity boundaries**. The upper window shows the number of mass peaks matched in each of the two mixes (no intensity boundaries, all peaks included). The bottom left window shows Peaks Matched per Compound (exact number and %) for the two mixes. The compound correspondence lists on the bottom right show compounds that are related, since peaks that belong to them were matched to each other.

As is apparent in figure 5, which shows the results of the matching process between the two mixes, many mass peaks from both mixes are not matched. This is surprising especially for the first mix, in which ideally most of the peaks should be matched to the corresponding peaks of the second mix, since they both contain the basic peptide mix.

The compound stats windows (figure 5) can provide more information about the peaks that are matched per compound in each mix, along with the compound correspondence between the two mixes (two compounds are considered matched, when mass peaks from the first are matched to mass peaks from the second). The first two (vertically) windows show the actual number of peaks per compound that were matched in each mix. More useful are the other two plots, which show the percentage of peaks in every compound that were matched. In the first mix, the majority of the compounds have peaks that are matched to the second mix, as should be expected, but the matching percentage is pretty low and vary between 30–60%. The compounds that appear to be unmatched in the first mix are some low intensity compounds that exist in the beginning and the end of the chromatogram, and also in the time space that the added compounds appear in the second mix, which have much higher intensity. By examining the plot for the second mix, it can be noticed that almost two thirds of the compounds have matched peaks and their matching percentage is between 20–80%. The two white regions correspond to compounds 4–7 and 15–21. These compounds have mass peaks that appear only in the second mix, so their origin is from the added peptide mix. The compound number correspondence between the two mixes is seen in the two lists. Clicking in a line of those lists highlights the selected compounds in the two chromatogram windows of the dual view.

Finally, the combinatory quantification tables can be seen by clicking on the results table button. The time values that appear in the quantification tables are the initial (before the shifting process) values.

**Figure 6:**
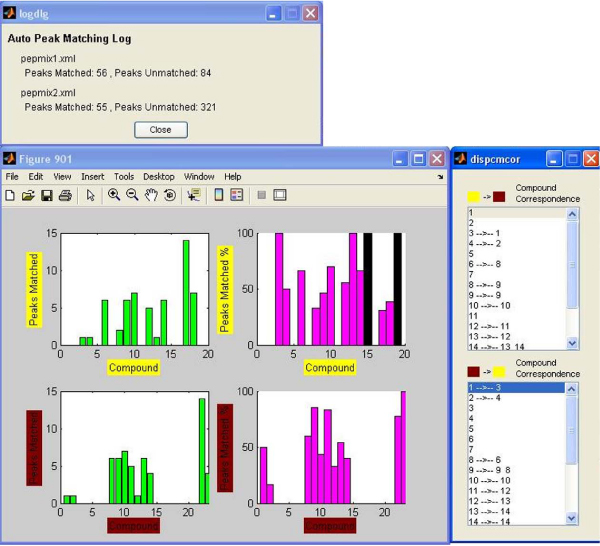
**Results of the matching process – 10000 intensity minimum cutoff**. Same as in figure 5 but with a 10000 intensity minimum cutoff.

**Figure 7:**
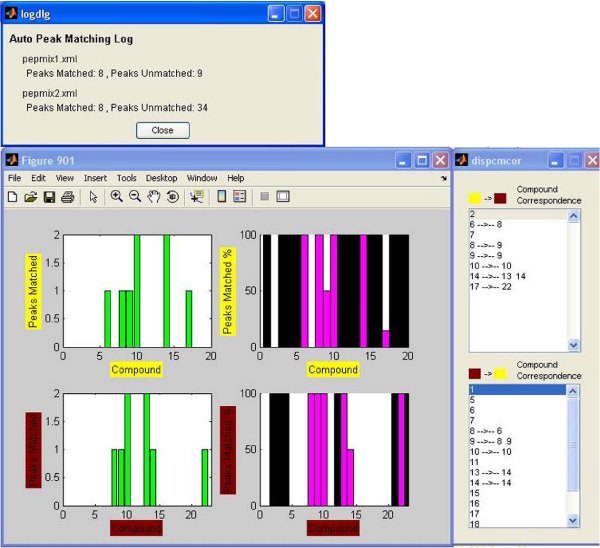
**Results of the matching process – 50000 intensity minimum cutoff**. Same as in figure 5 but with a 50000 intensity minimum cutoff.

In this example, the peak matching procedure was repeated two more times by setting two different mass intensity minimums in the contour plots, in order to take into consideration only mass peaks with high intensity. This was done by returning to the main program window and refreshing the contour plots, in order to exclude mass peaks that had intensity lower than 10000 and 50000 each time. In the first case, the peaks that were left in the two mixes were 140 and 376, and in the second case, only 17 and 42 peaks had intensity above 50000. After creating the new contours, the same shift vectors were used and the peak matching process was repeated. The results can be seen in figure 6 and figure 7 respectively. The black columns in the compound percentage plots represent compounds that had no mass peaks with intensity greater than the minimum. It can be noticed that by keeping only high intensity peaks, the matching results are better, meaning that the percentage of the matched peaks is raised. Some slight differences in the compound correspondence between the first comparison and the other two are due to mass intensity differences that exist between same peaks of the two mixes (peaks from one mix may have intensity lower than the boundary, while their matched peaks from the other mix have higher).

### Comparison with equivalent tools

Similar software applications to 'Brukin2D' have been developed by other research groups. Two of these applications are briefly discussed below. The first is 'Pep3D' [7], developed by The Institute for Systems Biology, which displays LC-ESI-MS data in a two-dimensional (retention time vs m/z) density plot, similar to the 'Brukin2D'. 'Pep3D' provides further functionality by allowing users to display precursor ions selected for CID and peptides successfully identified in the MS analysis. The CID boxes and peptide boxes have embedded common gateway interface (CGI) links allowing display of the corresponding CID spectrum and its peptide identification [2]. 'Ped3D' is available as part of the Trans-Proteomic Pipeline (TPP), which is a collection of integrated tools for MS/MS proteomics. The main feature of 'Ped3D' is the ability to include MS/MS data, acquired from SEQUEST or PeptideProphet, as annotations in the plot, and it lacks all functionality relevant to analyzing and comparing the mass peaks on the 2D plots. As such, 'Brukin2D' and 'Pep3D' can be considered and used as complementary applications. The second application is 'MSight' [8], developed by The Proteome Informatics Group. 'MSight' is a tool developed for the representation of mass spectra along with data from a separation step (such as LC-MS or SDS-MS). The 'MSight' interface and functionalities are based on the ImageMaster 2D gel image analysis system. 'MSight' handles data generated from many different mass spectrometers as well as generic data formats. MS/MS identifications can also be imported from the 'Phenyx' tool [9]. This software provides plenty capabilities, like annotation of peaks in the plots, three-dimensional view of a region, alignment and superposition of different MS runs as well as warping of plots, based on manually inserted landmarks [10]. The functionality of 'Brukin2D' is very similar to that of 'Msight'. Both applications visualize data in the same manner and provide the ability to superimpose MS runs and align them accordingly in order to eliminate known retention time errors from the chromatograph. Two main advantages of 'MSight' is the support for data acquired by many different instruments, including Bruker Daltonics, Applied Biosystems, Waters and ThermoFinnigan, and the ability to incorporate MS/MS annotations into the plot, in a similar way to that of 'Pep3D'. On the other side, mass peak identification and comparison is not yet supported by this application. These features will be introduced on the next version of the software, as mentioned on the 'MSight' website. Other relevant tools that must be mentioned are 'msInspect' [11] and 'MZmine' [12,13]. A more detailed description of all the above applications can be found in Veltri's work [14].

While 'Brukin2D' lacks the basic annotation functions of the aforementioned tools and it has been tested to work only with data exported from Bruker 'DataAnalysis', it provides a number of unique features to the user: (1) The chromatogram of an analysis can be imported and used in order to navigate among the different compounds. (2) Zooming is fast and unrestricted with no loss of data. (3) Besides warping and superimposing two plots, 'Brukin2D' includes a procedure for automatic matching of two contour plots, and the ability to view statistics calculated from the matching process results. Table 1 includes a summary of features that are supported by 'Brukin2D', 'MSight' and 'Ped3D'.

**Table 1:** Summary of supported features for Brukin2D, MSight and Pep3D

	**Brukin2D**	**MSight**	**Ped3D**
**Data types supported**	XML/mzXML	mzXML/mzDATA/native data files	mzXML/INTERACT data files
**Visualization of multiple MS runs**	Yes	Yes	Yes
**Support annotations (CID, peptides)**	No	Yes	Yes
**Mass peak identification**	Yes	No*	No
**Alignment and superposition of plots**	Yes	Yes	No
**Peak matching between MS runs**	Yes	No*	No
**Availability**	Free	Free	Free

*: According to the MSight website, these features will be available in the second version of the application.

## Discussion

The major concern in LC-MS is coping with the amount, redundancy and diversity of the exported data. A long way is still ahead of us in order to find quick, automated and definite procedures to evaluate these data. Better pre-processing and post-processing of data can lead to more reliable and meaningful results.

Based on our experience, tools like 'Brukin2D' appear to be valuable assets, when it comes to finding an easy way to evaluate LC-MS high-throughput data. Experience of the user in LC-MS is a major issue and should be considered necessary at least for the time being. The utilization and feedback on these tools from experienced scientists will result in the improvement of the software, which strives to facilitate data interpretation in a biologically meaningful way.

### Future work

Ongoing work and future plans include the extension of this application in order to be compatible with other LC-MS instrumentation software, primarily by making use of exported xml data, while considering the incorporation of support for native data files. Furthermore, effort is being done to provide better annotation capabilities and implement an automated chromatogram aligning algorithm, so as to replace the manually inserted shift vectors and avoid errors that can infiltrate the procedure through incorrect observations. Finally, rewriting the application in a more common programming language is considered in order to make the application more machine/software independent and less liable to memory issues, which for now pose a problem to the increasing number of plots that can be loaded into the application and compared.

## Conclusion

In this project, we developed a toolkit for easy visualization of the results from a LS-MS analysis, by using a 2D contour plot. The appropriate application and GUI for importing the LS-MS compound data from the Bruker 'DataAnalysis' program and plotting the contour with the desired parameters, were developed. In addition, the application incorporates the necessary tools in order to compare two contour plots, and consequently the corresponding peptide mixes from different chromatograph runs and find the differences in the compound mass spectra and the chromatograms. Thus, 'Brukin2D' can be used both as a visualization tool for LC-MS data and as a comparison tool, in order to find and evaluate in an easy and quick way the different mass peaks from multiple MS runs. Contour plots can be customized through a variety of parameters, in order to include the desired data and achieve the desired level of detail. All contour plots and corresponding data can be exported in standard formats for further usage, along with results from the comparison process. The tool and its source code are freely available, as well as the sample data files and the application manual. Ongoing work involves making the application instrument and OS (operating system) independent.

## Availability and requirements

*Project Name*: Brukin2D

*Project Home Page*: 

*Operating System(s)*: Cross-platform (dependent on Matlab availability, tested on Windows XP)

*Programming Language*: Matlab 7.4

*Other Requirements*: none

*License*: license-free

*Any restrictions to use by non-academics*: none

## Competing interests

The authors declare that they have no competing interests.

## Authors' contributions

MB and SK conceived and supervised the project. DT designed and implemented the software. SK, PZ, GL, AV and DT participated in the preparation of the manuscript. All authors have read and approved the final version of the manuscript.
